# Amoxicillin rash in patients with infectious mononucleosis: evidence of true drug sensitization

**DOI:** 10.1186/1710-1492-11-1

**Published:** 2015-01-09

**Authors:** Katinka Ónodi-Nagy, Ágnes Kinyó, Angéla Meszes, Edina Garaczi, Lajos Kemény, Zsuzsanna Bata-Csörgő

**Affiliations:** Department of Dermatology and Allergology, University of Szeged, Albert Szent-Györgyi Medical Center, Korányi fasor 6, Szeged, 6720 Hungary; Dermatological Research Group of the Hungarian Academy of Sciences, University of Szeged, Szeged, Hungary

**Keywords:** Amoxicillin, Sensitization to antibiotics, Infectious mononucleosis, Drug tests

## Abstract

**Background:**

It hasn’t been clearly understood yet whether sensitization to antibiotics, the virus itself or transient loss of drug tolerance due to the virus, is responsible for the development of maculopapular exanthems following amoxicillin intake in patients with infectious mononucleosis. We aimed to examine whether sensitization to penicillin developed among patients with skin rash following amoxicillin treatment within infectious mononucleosis.

**Methods:**

Ten patients were investigated for drug sensitization by lymphocyte transformation test and six patients were further tested by prick-, intradermal and patch tests employing the penicillin’s main antigens.

**Results:**

Lymphocyte transformation test showed negative results with amoxicillin, while one patient had positive reaction to cefixime. Six patients with suspected sensitization to amoxicillin were then investigated by *in vivo* tests. Prick tests were negative in all six patients, but the intradermal tests showed positive reactions in four patients.

**Conclusions:**

Our data demonstrate that *in vitro* testing is not sensitive enough in determining drug sensitization to penicillin. *In vivo* tests should be performed to detect sensitization and indeed with skin tests our results confirmed that sensitization to aminopenicillin may develop within infectious mononucleosis.

## Introduction

**Figure 1:**
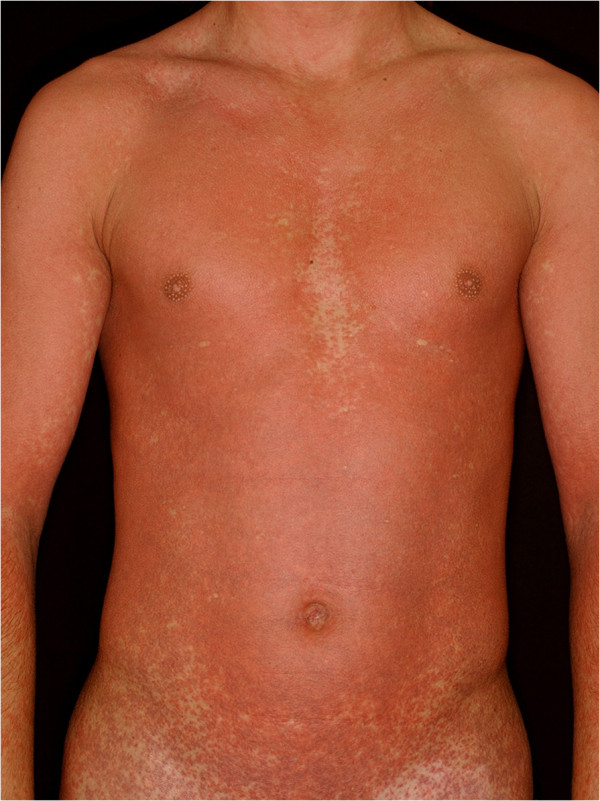
**Amoxicillin rash in a patient with infectious mononucleosis (patient 4).** The cutaneous eruptions developed a few days after the initiation of the antibiotic therapy. In severe cases the progressive maculopapular exanthems turn into erythroderma.

Infectious mononucleosis (IM) is an acute disease mostly caused by a widespread human γ-herpes virus, the Epstein-Barr virus (EBV) or a human β-herpes virus, the cytomegalovirus. The primary infection appears predominantly in children, adolescents and young adults [1]. Symptoms start with a prodromal phase including subfebrility, malaise, arthralgia and myalgia, like any common upper respiratory tract infection [2]. The classic features, fever, tonsillopharyngitis, lymphadenopathy, leukocytosis and hepatosplenomegaly, are helpful in differentiation from bacterial infection. Skin eruptions may develop during the infection. These eruptions are maculopapular exanthems, morbilliform eruptions on the whole body, in severe cases the progressive skin reaction turns into erythroderma (Figure 1). A severe cutaneous reaction such as erythema multiforme is exceedingly rare, although possible manifestation [3]. The skin symptoms may develop due to the viral infection, however, these patients often use antibiotics and it is also well-known that viral infections enhance the risk of drug allergic reactions [4, 5].

Eosinophil rich maculopapular exanthems occurring in mononucleosis rash are considered to be delayed type hypersensitivity reactions, in which Th2 T cells are activated and secrete IL-4, IL-5 and IL-13 that leads to eosinophilic inflammation. However secretion of IgE and IgG4 by B-cells accompanies the reaction, connecting the delayed reaction to immediate type I reaction [6]. We aimed to examine whether sensitization to penicillin developed among patients with cutaneous rash following amoxicillin treatment within IM.

## Findings

### Materials and methods

#### Patient selection

At the Department of Dermatology and Allergology, University of Szeged in Hungary among those patients who were treated between 2002 and 2012, ten young adults (5 men and 5 women, median age 22.9, range 15–35 years) with the diagnosis of IM, confirmed by EBV serological assay (specific IgM and IgG antibodies), associated with generalized maculopapular eruptions were examined for sensitization to antibiotics. All of these patients underwent antibiotic therapy prior to the appearance of skin eruptions. In all cases the antibiotic was amoxicillin/clavulanic acid, in 2 cases in addition to penicillin the patients were given clarithromycin or cefixime as well. Although clinically the skin symptoms of Drug Reaction with Eosinophilia and Systemic Symptoms (DRESS) can be indistinguishable, DRESS have strict criteria, which were not met in our patients [7].

#### In vitro tests: lymphocyte transformation test (LTT)

We examined mononucleosis infectiosa patients with a history of penicillin intake, with an *in vitro* method, the LTT, 1–1.5 months after the cessation of skin eruptions. The LTT was performed to determine T-cell proliferation as an indicator of drug sensitization as described previously [8, 9], with minor modifications. Briefly, peripheral blood mononuclear cells were isolated from heparinized peripheral blood and cultured under defined conditions with various concentrations of the suspected drugs (100 μg/ml and 10 μg/ml dilutions), in our cases with amoxicillin, amoxicillin/clavulanic acid, penicillin and cefixime [10]. We evaluated cell growth in the cultures. Cell growth was measured by using a colorimetric assay and an automatic microplate scanning spectrophotometer. The assay depends on the reduction of tetrazolium salt (MTT: 3-(4,5-dimethylthiazol-2-yl)-2,5-diphenyltetrazolium bromide) by living cells, to form a blue insoluble formazan product [11]. During the investigation we used the spontaneous cell growth as negative control, while the phytohaemagglutinin-stimulated cell culture served as positive control. The results were recognized as positive, if the drug stimulated cell numbers were at least twice higher that the negative control’s (stimulation index >2).

#### In vivo test: skin tests

**Figure 2:**
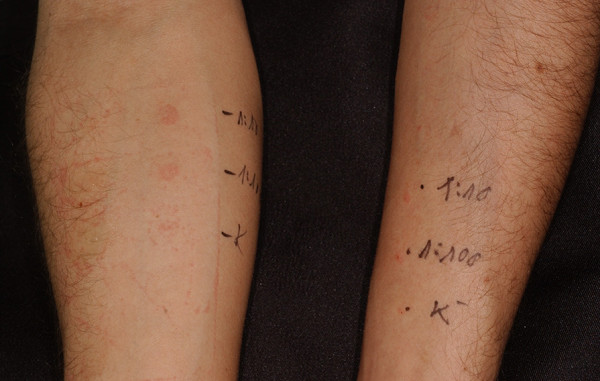
**Positive cutaneous response (Penicillin allergenic determinants (DAP) ® test).** The *in vivo* cutaneous investigation was continued with intradermal testing, if the prick tests resulted in negative response at different dilutions. Skin tests were performed using penicillin’s main antigens, *major determinants and* (PPL) and m*inor determinant mix* (MDM). Cutaneous tests were started with major determinants, the negative control was saline solution. In this case we recognized positive skin reaction to MDM at 1:100 and 1:10 dilutions, which verified the development of sensitization (patient 4).

We performed *in vivo* cutaneous tests using penicillins in patients with negative LTT to amoxicillin. The remaining patients refused to consent to testing. Prick, intradermal and patch tests were performed using penicillin’s main antigens: *major determinant* benzylpenicilloyl poly-L-lysine (PPL), m*inor determinant mix s*odium benzylpenicillin, benzylpenicilloic acid, sodium benzylpenicilloate (MDM) from Diater Laboratorios (Penicillin allergenic determinants (DAP) ® test) [12, 13]. We followed the investigation protocol given by the manufacturer [14]. Cutaneous tests were started with major determinants (Figure 2). If the prick tests at different dilutions were negative, the testing was continued with intradermal and then patch tests. Each prick and intradermal tests were read once 20 minutes elapsed since their application. Tests results were also read at 24, 48, 72 and 96 hours for detecting delayed reactions. Patch tests were performed using the powdered culprit drug mixed into vaseline (1:1). Allergens were applied to the upper back in individual round chambers (Curatest®, Spiromed Ltd.). Readings were performed at 48, 72, 96 hours and 7 days [15, 16]. Although skin rashes occurring in mononucleosis are likely delayed type reactions, we performed immediate reading, because clinical history cannot always be trusted, patients will report a delayed reaction which is in fact an immediate one.

### Results

Among those patients who were treated at our clinic between 2002 and 2012 10 patients (median age 22.9, range 15–35 years) with IM and maculopapular rash were examined by drug allergy tests. All patients took antibiotics before the appearance of skin symptoms. In all ten patients *in vitro* LTTs were performed with the suspected drugs. Amoxicillin/clavulanic acid enhanced drug-specific response in none of the cases. Increased lymphocyte proliferation was found with one peripheral blood sample after incubation with cefixime. Six out of the 10 patients with suspected sensitization to amoxicillin and negative LTT results were further investigated *in vivo* by prick, intracutaneous and patch testing. All six patients showed negative responses with prick tests. The intradermal tests resulted in positive reactions in four subjects. Patch tests were performed after negative prick and intracutaneous testing with negative results in the remaining two patients (Table 1).

**Table 1:** **True sensitization to amoxicillin examined by** ***in vivo*** **cutaneous tests**

Patient	Age (years)	Gender	Culprit drug	LTT results	Prick test results	Intradermal test results	Patch test results
**1**	15	female	amoxicillin/clavulanic acid	Negative	Negative	**PPL 1:100 and 1:10 Positive**	Not performed
**2**	19	female	amoxicillin	Negative	Negative	**MDM undiluted Positive**	Not performed
**3**	29	female	amoxicillin/clavulanic acid	Negative	Negative	**PPL 1:10 Positive**	Not performed
**4**	23	male	amoxicillin/clavulanic acid	Negative	Negative	**MDM 1:100 and 1:10 Positive**	Not performed
5	35	male	amoxicillin/clavulanic acid	Negative	Negative	Negative	Negative
6	24	female	amoxicillin/clavulanic acid	Negative	Negative	Negative	Negative
7	21	male	amoxicillin	Negative	Not performed	Not performed	Not performed
8	20	female	amoxicillin/clavulanic acid	Negative	Not performed	Not performed	Not performed
9	16	male	amoxicillin/clavulanic acid	Negative	Not performed	Not performed	Not performed
10	27	male	amoxicillin/clavulanic acid; cefixime	Positive: cefixime	Not performed	Not performed	Not performed

PPL: *major determinant:* benzylpenicilloyl poly-L-lysine.

MDM: m*inor determinant mix: s*odium benzylpenicillin, benzylpenicilloic acid, sodium benzylpenicilloate.

**Bold text:** Verified sensitization to penicillin.

Tests were done in the following chronology: LTT → Prick test *(non-diluted PPL)* → Intradermal test *(1:100 dilution of PPL, 1:10 dilution of PPL, non-diluted PPL)* → Prick test *(non-diluted MDM)* → Intradermal test *(1:100 dilution of MDM, 1:10 dilution of MDM, non-diluted MDM)* → Patch test *(culprit drug).*

It is important to notice that the *in vivo* investigations were carried out at least six month after the disappearance of the eruptions which lead us to think that drug sensitization developed instead of a transient loss of tolerance, a transient Th-1 lymphocyte-mediated delayed type hypersensitivity reaction to the medication as discussed in the literature [17, 18].

## Discussion

The development of skin rash following amoxicillin intake in patients with IM is quite frequent among beta-lactam-induced adverse drug reactions [19]. These eruptions are maculopapular exanthems. The exact mechanism behind them is unclear. It is not well explained yet, whether a true allergic drug reaction, virus-dependent rash or transient loss of drug tolerance due to the virus is responsible for the symptoms. The rash may be due to the viral infection itself, the incidence of skin eruption development in acute IM is 4.2-13% without drug intake, but often these patients are put on antibiotics, frequently amoxicillin, and the rash appears a few days after the initiation of the antibiotic therapy [20]. Following amoxicillin intake within acute IM the incidence of skin reactions ranges between 27.8% and 69%, while in children, morbilliform skin eruptions nearly always develop following amoxicillin intake within acute IM [4, 21, 22].

Our aim was to find out whether true amoxicillin sensitization developed for aminopenicillin among our patients. Evidence shows in recently published literature that the development of allergic reaction for aminopenicillin during a florid viral infection is definitely more prevalent as it was believed previously [4, 5]. Although Renn et al. earlier demonstrated true sensitizations to amoxicillin in three patients with IM and clear history of amoxicillin intake with positive proliferative responses, we further investigated this phenomenon to provide more evidence. Our results add additional evidence that indeed in such patients drug sensitization develops during the infection. According to the current recommendations in drug allergy, positive skin tests for beta-lactam antiobiotics (following standardized reading) do not require challenge tests, to confirm clinical relevance. Skin tests are validated, while LTT is not. We can not explain the negative results of LTT in all of these cases to penicillins. The two patients with negative *in vitro* and *in vivo* test results need to be further investigated by performing cutaneous tests with the culprit drug and if this was negative a drug provocation test should be applied in order to prove that the patient did not developed penicillin allergy. In this work our primary aim was to demonstrate that true sensitization can occur within mononucleosis infectiosa patients suffering from amoxicillin rash.

With this investigation we would like to further emphasize the importance of allergy examination in patients with generalized skin lesions after penicillin intake in IM, to verify whether true sensitization developed.

## Conclusions

Our data demonstrate that *in vitro* testing is not sensitive enough in determining drug sensitization for penicillin in patients who develop skin symptoms during mononucleosis infection. *In vivo* tests should be performed to detect sensitization and indeed with skin tests our results confirmed that sensitization to aminopenicillin may develop within infectious mononucleosis.

## Abbreviations

IM: Infectious mononucleosis
EBV: Epstein-barr virus
DRESS: Drug reaction with eosinophilia and systemic symptoms
LTT: Lymphocyte transformation test
MTT: 3-(4,5-dimethylthiazol-2-yl)-2,5-diphenyltetrazolium bromide
PPL: Benzylpenicilloyl poly-L-lysine
MDM: Minor determinant mix
DAP ®: Penicillin allergenic determinants.
